# Characterization, Biocontrol, and Fungicide Sensitivity of *Macrophomina phaseolina* Isolates Associated with Charcoal Rot of Sesame in Northern Sinaloa, Mexico

**DOI:** 10.3390/jof12070464

**Published:** 2026-06-24

**Authors:** Elizabeth García-León, Víctor Hugo Aguilar-Pérez, Juan Manuel Tovar-Pedraza, Guillermo Márquez-Licona, Hugo Beltrán-Peña, Moisés Camacho-Tapia, Luis Alfonso Aguilar-Pérez, Alma Rosa Solano-Báez

**Affiliations:** 1 Campo Experimental Valle del Fuerte, Instituto Nacional de Investigaciones Forestales, Agrícolas y Pecuarias, Juan José Ríos 81110, Sinaloa, Mexico; garcia.elizabeth@inifap.gob.mx; 2 Facultad de Agricultura del Valle del Fuerte, Universidad Autónoma de Sinaloa, Juan José Ríos 81110, Sinaloa, Mexico; hugoapp1804@gmail.com (V.H.A.-P.); hugobeltran@favf.mx (H.B.-P.); 3 Centro de Investigación en Alimentación y Desarrollo, Subsede Culiacán, Laboratorio de Fitopatología, Culiacán 80110, Sinaloa, Mexico; juan.tovar@ciad.mx; 4 Instituto Politécnico Nacional, Centro de Desarrollo de Productos Bióticos, Yautepec 62731, Morelos, Mexico; gmarquezl@ipn.mx; 5 Departamento de Parasitología Agricola, Universidad Autónoma Chapingo, Texcoco 56230, Estado de Mexico, Mexico; moises.camachotapia@gmail.com; 6 Facultad de Ciencias Biológicas y Agropecuarias, Universidad Veracruzana, Amatlán de Los Reyes 94945, Veracruz, Mexico; luiaguilar@uv.mx

**Keywords:** *Sesamum indicum*, pathogenicity, *Trichoderma*, antagonisms, fungicide sensitivity

## Abstract

*Macrophomina phaseolina* is a plant-pathogenic fungus that causes charcoal rot in sesame crops, which is the most significant disease affecting this crop worldwide. In Mexico, the interaction between *M. phaseolina* and sesame has been poorly studied. Therefore, this research aimed to characterize *Macrophomina* spp. isolates from diseased sesame roots in northern Sinaloa, Mexico, using morphological, molecular, and pathogenic methods. It also assessed the in vitro effectiveness of biocontrol agents and chemical fungicides. Six isolates of *Macrophomina* were identified through morphology, species-specific *tef1-α* primers, and phylogenetic analysis of DNA sequences (ITS + *tef1-α*), confirming their identity as *M. phaseolina*; all isolates proved to be pathogenic. Antagonism assays with *Trichoderma* spp. showed statistically significant differences. *Trichoderma* isolates inhibited mycelial growth by up to 63% against *M. phaseolina*. In fungicide sensitivity tests, *M. phaseolina* isolates showed EC_50_ values ranging from 0.002–0.123, 0.049 to 1.397 and 0.029 to 0.539 mg L^−1^ for thiophanate-methyl, tebuconazole, and pyraclostrobin, respectively. In summary, *Trichoderma* spp. isolates and the tested fungicides warrant further research as potential strategies to manage *M. phaseolina* in sesame fields.

## 1. Introduction

Sesame (*Sesamum indicum* L.: Pedaliaceae) is an oilseed crop known for its high oil content, ranging from 45 to 50%, which makes it highly nutritious and versatile for industry [1]. In Mexico, sesame represents a profitable crop option due to its low production costs, minimal water requirements, the incorporation of non-shattering cultivars suitable for direct mechanical harvesting, and stable market prices [2]. In 2024, a total of 64,318 hectares of sesame were cultivated in Mexico, with the main producing states being Sinaloa (19,884 ha), Guerrero (16,015.39 ha), Michoacán (10,156 ha), Oaxaca (8380.50 ha), and Chiapas (7572.80 ha), which together account for more than 90% of the national production [3]. In Sinaloa, sesame cultivation is divided between rainfed and irrigated systems, depending on water availability. Irrigated sesame is primarily grown in the municipality of Ahome, while rainfed sesame is cultivated in Choix, El Fuerte, Mocorito, Salvador Alvarado, Sinaloa de Leyva, Guasave, Culiacán, and Badiraguato. In 2024, the municipality of Sinaloa de Leyva recorded the largest planted area (10,935 ha), with an average yield of 0.44 t ha^−1^ [3].

Sesame production is severely affected by diseases caused by fungal pathogens such as *Cercospora sesami* [4], *Fusarium oxysporum* [5], *Agroathelia rolfsii* [6], *Colletotrichum truncatum* [2], *Alternaria* spp. [7], and *Sclerotinia sclerotiorum* [8]. The presence of these fungal pathogens causes yield losses and reduces seed quality for both planting and industrial use [9,10]. However, the most economically important disease of sesame worldwide is stem and root rot caused by *Macrophomina phaseolina* [11,12], although this pathosystem has not been studied in Mexico. Soilborne pathogen infections manifest as root rot, wilting, and damping-off symptoms [13]. Understanding the genetic and pathogenic variability of pathogen populations is essential for implementing effective disease management strategies, such as the use of tolerant or resistant cultivars [14].

*Macrophomina phaseolina* is a necrotrophic soilborne fungus belonging to the family Botryosphaeriaceae, with a global distribution and a host range of nearly 500 economically important plant species, including common bean (*Phaseolus vulgaris*), maize (*Zea mays*), potato (*Solanum tuberosum*), chili pepper (*Capsicum annuum*), chickpea (*Cicer arietinum*), sorghum (*Sorghum bicolor*), peanut (*Arachis hypogaea*), soybean (*Glycine max*), and cotton (*Gossypium hirsutum*) [15,16,17,18]. Species identification within the *Macrophomina* genus based solely on morphological and cultural traits is considered insufficient [9]. Due to the limitations of conventional methods, molecular tools have been implemented, including species-specific primers [19,20] and phylogenetic analyses using DNA sequences from various molecular markers, such as the internal transcribed spacer (ITS) region, and gene fragments of calmodulin (*cal*), translation elongation factor 1-alpha (*tef1-α*), and β-tubulin (*tub2*) [10].

Chemical fungicides are the primary method used to manage soilborne pathogens such as *M. phaseolina*, with active ingredients from different chemical groups, including thiophanate-methyl (Methyl Benzimidazole Carbamates, MBC), pyraclostrobin (Quinone outside Inhibitors, QoI), and tebuconazole (Demethylation Inhibitors, DMI). However, concerns regarding environmental impact, human health risks, and increased production costs associated with fungicide use have led to growing interest in biological alternatives for disease management. Biological control agents have been adopted as environmentally friendly tools that leverage ecosystem interactions to balance productivity, economy, and sustainability [21,22,23]. In sesame, biological control agents such as *Trichoderma* spp. and *Bacillus subtilis* have been used to manage *F. oxysporum* and *M. phaseolina* in India and Egypt [24,25].

During the 2020 and 2021 spring-summer growing seasons in Sinaloa (Mexico), commercial sesame fields in the municipalities of Ahome, El Fuerte, Salvador Alvarado, and Mocorito exhibited high incidences (30–100%) (unpublished data) of root and stem rot symptoms. The incidence was assessed by evaluating the percentage of plants showing characteristic symptoms. To date, there is no precise record of the fungi associated with the death of sesame plants. Therefore, the objectives of this study were to characterize fungal isolates associated with sesame root rot and to evaluate the antagonistic potential of *Trichoderma* spp. and the pathogen’s in vitro sensitivity to fungicides.

## 2. Materials and Methods

### 2.1. Isolation, Purification, and Preservation of Fungal Isolates

**Figure 1:**
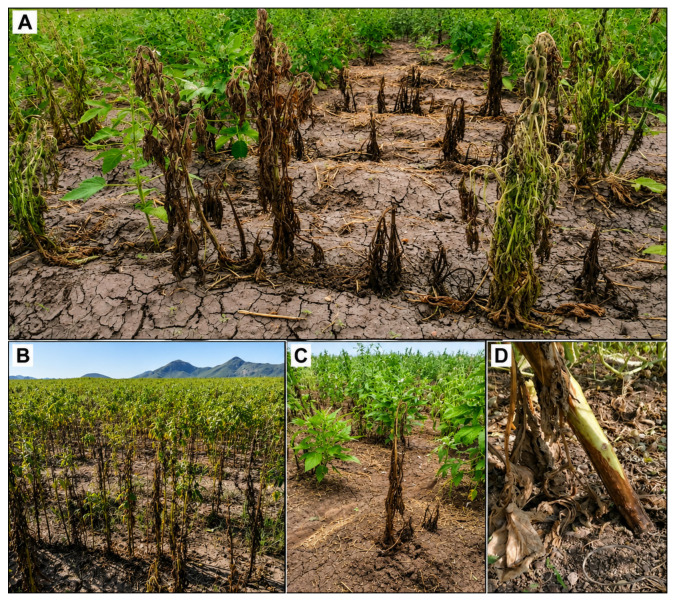
Charcoal rot symptoms caused by *Macrophomina* sp. in sesame plants from commercial fields in Sinaloa, Mexico. (**A**,**B**) Fields showing a high incidence of the disease. (**C**) Symptomatic plant (foreground) and asymptomatic plant (background) of the same age. (**D**) Stem base with symptoms of ascending necrosis and brown to black lesions near soil level.

During the growing seasons (May–August) 2020 and 2021, 30 plants showing stem and root rot symptoms (Figure 1) were collected from 8 and 15 commercial fields, respectively, in the state of Sinaloa, Mexico. Mean monthly temperatures during the sampling periods ranged from 26.2 to 38.8 °C. These fields were distributed in the municipalities of Ahome, Choix, El Fuerte, Mocorito, Salvador Alvarado, and Sinaloa de Leyva, and the cultivars grown included Paraguayo, Dormilón, Breve Doble, and Pachequeño.

Samples were transported to the Plant Pathology Laboratory at the Universidad Autónoma de Occidente and processed for fungal isolation. Fungal isolates were obtained following the methodology described by Crous et al. (2009) [26]. From the margin between healthy and diseased tissue, 5 × 5 mm sections were cut, surface-disinfected in 2% sodium hypochlorite (NaClO) for 2 min, rinsed three times with sterile distilled water, and dried on sterile absorbent paper. The disinfected tissue sections were transferred to Petri plates containing potato dextrose agar (PDA; BD Bioxon^®^, Cuautitlán Izcalli, Estado de Mexico, Mexico) and incubated at 25 °C for four days. Pure cultures were obtained using the single-hyphal-tip technique on water agar (WA).

The axenic isolates were deposited in the culture collection of the Phytopathology Laboratory at the Center for Research in Food and Development (CIAD), Culiacán, Sinaloa, Mexico, under codes CCLF670–CCLF675. Isolates were preserved in cryovials containing 10% glycerol at −80 °C.

### 2.2. Morphological Identification

The mycelial growth rate of each purified isolate was measured by transferring 5-mm-diameter mycelial plugs to the center of Petri plates containing PDA and incubating them at 25 °C in continuous darkness. Colony diameter was measured every 24 h along polar and equatorial axes using a digital caliper (Truper, Mexico), and the radial growth rate was calculated using the formula developed by Zervakis et al. (2001) [27]. Three replicates were used per isolate, and the assay was conducted twice.

Cultural characteristics such as colony color, mycelial type, and microsclerotia production were evaluated on PDA, water agar (WA), and pine needle agar (PNA). Microscopic traits, including microsclerotia diameter (n = 50) and hyphal pigmentation, were examined on PDA and PNA media [28]. Growth rate and microsclerotia size data were subjected to Shapiro–Wilk’s test for normality, Levene’s test for homogeneity of variances, and mean comparisons were made using Tukey’s test with SAS 9.4 software (Statistical Analysis System, Cary, NC, USA).

### 2.3. Molecular Identification

Genomic DNA was extracted from 7-day-old colonies grown on PDA at 25 °C in continuous darkness. DNA extraction was performed using the DNeasy Plant Mini Kit (Qiagen, Germany) according to the manufacturer’s instructions. DNA integrity and concentration were assessed by spectrophotometry using a Nanodrop^®^ One (Thermo Fisher Scientific, Madison, WI, USA). DNA samples were adjusted to a concentration of 5 ng µL^−1^ with nuclease-free sterile water and stored at −20 °C until use. Polymerase chain reaction (PCR) was performed using ITS and species-specific *tef1-α* primers for *Macrophomina* as reported by Babu et al. (2007) [29] and Santos et al. (2020) [20], respectively. The ITS region was amplified with primers ITS5/ITS4 under the following conditions: initial denaturation at 95 °C for 5 min, followed by 25 cycles of 95 °C for 30 s, 56 °C for 60 s, and 72 °C for 120 s, with a final extension at 72 °C for 10 min and hold at 10 °C. Amplification of the *tef1-α* gene was carried out using primers MpTefF/MpTefR with the following thermal profile: initial denaturation at 94 °C for 120 s, followed by 30 cycles of 94 °C for 60 s, 63 °C for 30 s, and 72 °C for 60 s, with a final extension at 72 °C for 10 min and hold at 10 °C. All PCRs were performed in a final volume of 50 μL, containing: 25 μL of 2× PCR Master Mix (Promega Corporation, Madison, WI, USA), 2.5 μL of each primer (10 μM), 16 μL of nuclease-free water, and 4 μL of template DNA. PCR products were purified using the QIAquick Gel Extraction Kit (Qiagen, Germany) and sequenced bidirectionally by Macrogen Inc. (Seoul, Republic of Korea). The consensus sequences were deposited in the GenBank database under the accession numbers PV106184–PV106189 for ITS and PV113476–PV113481 for *tef1-α*.

### 2.4. Phylogenetic Analyses

Consensus sequences for both loci were compared to sequences available in the non-redundant GenBank database using BLASTn tool (https://blast.ncbi.nlm.nih.gov/Blast.cgi, accessed on 20 March 2025) to verify their identity. This information was used to trace a reference phylogeny [9], which helped in constructing the matrix for phylogenetic analysis, including the five described *Macrophomina* species. All reference isolate sequences were downloaded from the NCBI nucleotide database and aligned with our sequences using the MAFFT v7 online service [30]. The alignments of each locus were trimmed in MEGA 12 [31] and concatenated in Mesquite 4.01 [32]. For phylogenetic reconstruction, a Bayesian inference (BI) analysis was performed using MrBayes 3.2.7 [33], applying the GTR substitution model for both partitions, as chosen by the Akaike Information Criterion (AIC) in jModelTest 2.1.10 [34]. The Markov Chain Monte Carlo (MCMC) ran for 2 × 10^6^ generations to estimate posterior probabilities, sampling trees every 1000 generations and discarding 25% of trees during the burn-in. Additionally, a Maximum Likelihood (ML) phylogenetic analysis was conducted using RAxML 8.2.12 through the raxmlGUI 2.0 interface [35], with the GTR + G substitution model. Node support was evaluated with 1000 rapid bootstrap replicates. Phylogenies were visualized and edited with FigTree (http://tree.bio.ed.ac.uk/software/figtree/, accessed on 19 May 2025).

### 2.5. Pathogenicity Tests

The pathogenicity of the six *Macrophomina* isolates was evaluated on 21-day-old sesame plants of the Dormilón and Paraguayo cultivars. Sesame seeds were surface-disinfected and planted in sterilized substrate (Promix, Quakertown, PA, USA) in pots (35 × 20 cm). Each experimental unit consisted of a pot containing four plants, with four replicates per treatment arranged in a completely randomized design. Inoculation was performed by inserting wooden toothpicks colonized with *Macrophomina* mycelium and microsclerotia into lesions made at the base of the stem [36,37,38]. Meanwhile, the control plants were wounded with a sterile toothpick, omitting prior inoculation with microsclerotia. The virulence of *M. phaseolina* isolate on sesame plants was determined based on lesion size at 7 days post-inoculation (dpi). Disease severity and host reaction were recorded. The complete experiment was conducted twice.

### 2.6. Isolation and Identification of Antagonistic Fungi

Two isolates of *Trichoderma* spp. (FAVF675, FAVF676) were recovered from the rhizosphere soil of healthy sesame plants. For morphological analysis, macroscopic and microscopic characteristics were assessed, including the mycelial growth rate of each isolate, the type of mycelial growth, and colony pigmentation on natural PDA and Spezieller Nährstoffarmer Agar (SNA) media incubated at 25 °C in continuous darkness for seven days. For micromorphological analysis, the size and arrangement of phialides, the size and shape of conidia, and the presence or absence of chlamydospores were examined [26] . Three additional isolates of *Trichoderma* (H7, H21, and H27) were obtained from the fungal collection of the Phytopathology Laboratory of the Center for the Development of Biotic Products (CEPROBI) of the National Polytechnic Institute (Instituto Politécnico Nacional) in Yautepec, Morelos, Mexico. All isolates were characterized morphologically and by amplification of the ITS region. The nucleotide sequences generated in this study were deposited in GenBank under accession numbers PZ457791-PZ457795.

### 2.7. Antagonism Tests

In vitro antagonism was assessed using the dual culture technique. *Trichoderma* and *M. phaseolina* isolates used in the dual culture assays were obtained from 5-day-old PDA cultures prior to confrontation. A 5-mm mycelial plug of *M. phaseolina* was placed at one edge of a Petri plate containing PDA, and a 5-mm mycelial plug of the *Trichoderma* isolate was placed at the opposite edge. For the control, a 5-mm mycelial plug of *M. phaseolina* was placed on one side of the PDA medium without *Trichoderma*. Five replicates were included for each confrontation and control. Plates were incubated at 25 °C with a 12 h light/12 h dark photoperiod in a completely randomized design. The entire experiment was repeated twice. Mycelial growth of *M. phaseolina* was recorded every 24 h until the control isolate covered 95% of the plate surface. The percentage of mycelial growth inhibition (PMGI) was calculated using the formula: PMGI = (C − T) × 100/C, where C = radial growth (cm) of the pathogen in the control and T = radial growth (cm) in the presence of *Trichoderma* spp. [39].

### 2.8. In Vitro Fungicide Sensitivity

The in vitro fungicide sensitivity assay was conducted using commercial formulations of thiophanate-methyl (Cercobin 70 WG, BASF, Tokio, Japon), tebuconazole (Folicur 25 EW, Bayer CropScience AG, Monheim am Rhein, Germany), and pyraclostrobin (Headline 25 CE, BASF). Each fungicide was diluted in sterile distilled water to prepare a stock solution at 100 µg mL^−1^. From this stock, calculated volumes were added to sterile PDA medium cooled to 45 °C to obtain final concentrations of 0.005, 0.01, 0.05, 0.1, 0.5, 1, and 5 µg mL^−1^ for tebuconazole and pyraclostrobin, and 0.1, 0.5, 1.0, 5, 10, 50, and 100 µg mL^−1^ for thiophanate-methyl. Petri plates containing PDA without fungicides were used as controls. PDA plates with added fungicides were prepared 24 h before the test and kept in constant darkness. A 5-mm mycelial plug, taken from the margin of a 5-day-old colony, was placed in the center of each fungicide-amended plate. Control plates were inoculated with the same pathogen isolates without fungicide. Plates were incubated at 25 °C in continuous darkness until at least one control isolate covered 95% of the plate surface. Three replicates were used per fungicide concentration. For each isolate–fungicide combination and the control, colony diameter was measured perpendicular to the surface. Each assay was conducted twice. To block the alternative respiration of the fungus, a stock solution of salicylhydroxamic acid (SHAM, 99%; Sigma-Aldrich, St. Louis, MO, USA) was added to the medium containing pyraclostrobin to obtain a final SHAM concentration of 100 µg mL^−1^. SHAM was also added to control Petri dishes (without fungicide). The effective concentration inhibiting 50% of mycelial growth (EC_50_) for each isolate was calculated using a dose–response curve fitted to the data with a logistic-log regression model, as implemented in the RStudio software [40], utilizing the LL.3 model in the RStudio software R 2024.04.2 (Posit Software, PBC, Boston, MA, USA) [41].

### 2.9. Statistical Analysis

Statistical analyses were performed using RStudio software R 2024.04.2 (Posit Software, PBC, Boston, MA). Initially, data on the percentage of mycelial growth inhibition (PMGI) was analyzed using a one-way ANOVA, with *Trichoderma* isolates as fixed factors. Subsequently, a two-way ANOVA was performed to evaluate both *Trichoderma* and *M. phaseolina* isolates as factors, as well as their interaction.

Prior to analysis, normality and homogeneity of variances assumptions were verified using the Shapiro–Wilk and Levene’s tests, respectively. When the significant treatment effects were detected (*p* ≤ 0.05), means were compared using Tukey’s HSD test (α = 0.05). Results were expressed as mean ± standard error.

## 3. Results

### 3.1. Fungal Isolation

A total of 28 fungal isolates with colony characteristics typical of *Macrophomina* were obtained from diseased samples. Since no morphotypes were detected, six representative isolates (CCLF670, CCLF671, CCLF672, CCLF673, CCLF674, and CCLF675) were randomly selected and used for subsequent analyses.

### 3.2. Cultural and Morphological Characterization

**Figure 2:**
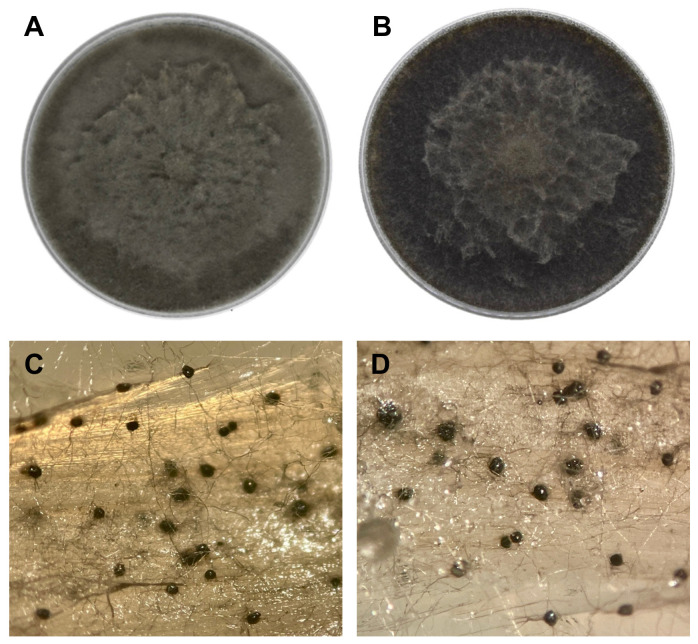
Colonies of *Macrophomina phaseolina* grown on potato dextrose agar (PDA) after 14 days of incubation. (**A**) Isolate CCLF671. (**B**) Isolate CCLF675. (**C**,**D**) Microsclerotia.

The mycelial growth rate of the six characterized isolates ranged from 18.90 mm day^−1^ (CCLF672) to 22.55 mm day^−1^ (CCLF671). On PDA medium, isolates exhibited floccose to dense cottony mycelial growth with olive-gray pigmentation that darkened after 14 days of incubation at 25 °C (Figure 2A,B). Microsclerotia formation on PDA (Figure 2C,D) and WA began five days after incubation, appearing as smooth, black, round to oval structures with an average diameter (Table 1) ranging from 62.6 µm (CCLF670) to 114.0 µm (CCLF674).

### 3.3. Molecular Identification

**Figure 3:**
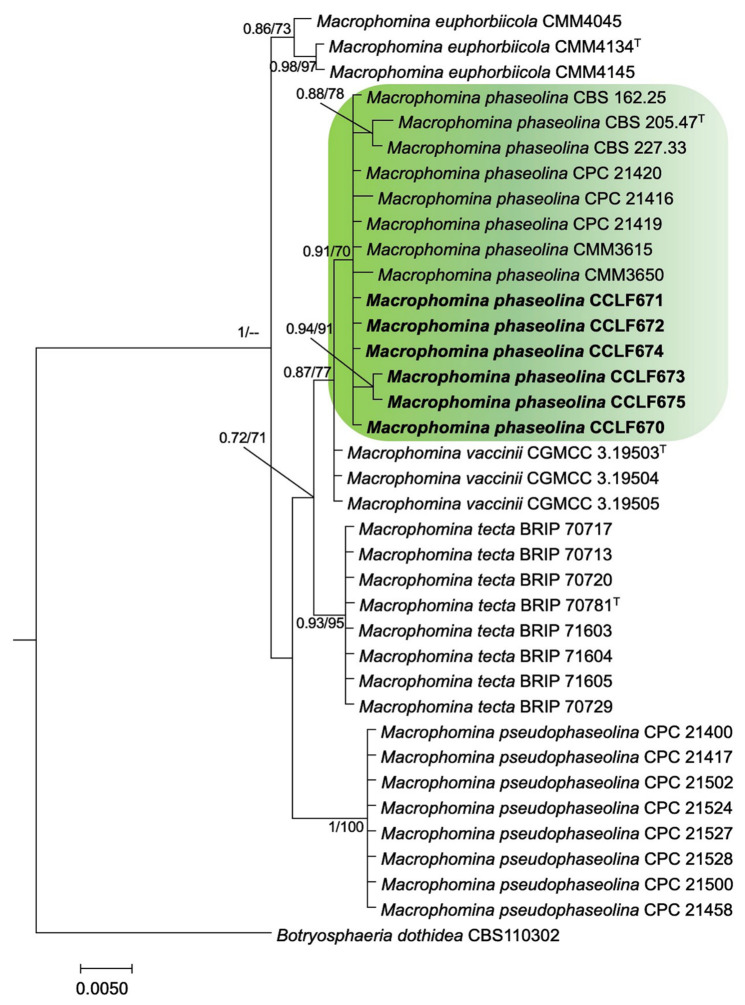
Phylogenetic tree constructed based on ITS, and *tef1-α* partial sequence data of taxa from the *Macrophomina* genus. Posterior probabilities (>0.7) and Bootstrap support (>70%) values for Bayesian Inference and Maximum likelihood are shown in the nodes. The tree was rooted to *Botryosphaeria dothidea* CBS 110302. Isolates of this study are shown in bold.

The ITS sequences of isolates CCLF670–CCLF675 (accession numbers PV106184–PV106189) showed 100% similarity to the ex-type isolate CBS 205.47 recovered from common bean (accession number KF951622). Meanwhile, the partial sequences of tef1-α with NCBI accession numbers PV113476–PV113481 showed 98% similarity to the ex-type isolate CBS 205.47 recovered from common bean (accession number KF951997). PCRs using ITS and *tef1-α* species-specific primers confirmed that all six isolates amplified exclusively with *Macrophomina phaseolina*-specific primers. This identification was further corroborated by phylogenetic analyses using Bayesian Inference and Maximum Likelihood methods based on concatenated ITS and *tef1-α* sequences, which placed it within the *M. phaseolina* clade with high posterior probability and bootstrap support (Figure 3).

### 3.4. Pathogenicity Assays

**Figure 4:**
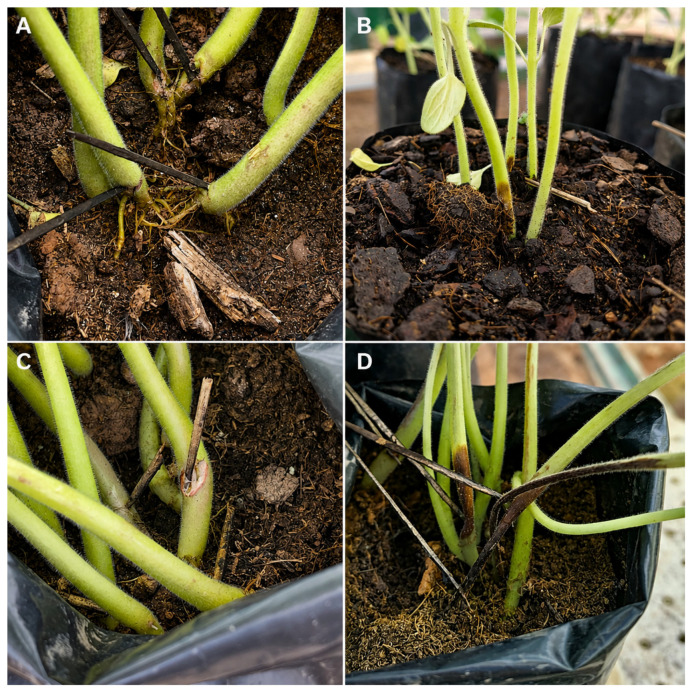
Symptoms developed in sesame plants (*Sesamum indicum*) 7 days after inoculation with *Macrophomina phaseolina* isolates. (**A**) Plants of the cultivar Paraguayo control, (**B**) Plants of the cultivar Paraguayo inoculated with isolate CCLF673. (**C**) Plants of the cultivar Dormilón control (**D**). Plants of the cultivar Dormilón inoculated with isolate CCLF674.

Sesame plants of cvs. Dormilón and Paraguayo inoculated with the six *M. phaseolina* isolates developed typical charcoal rot symptoms five days after inoculation (Figure 4B,D), including necrotic lesions with brown-to-black discoloration, stem base constriction, foliar chlorosis, and wilting. Control plants remained asymptomatic, although a small wound from mechanical damage was observed; the plants recovered, and no symptoms of infection were observed (Figure 4A,C).

Lesion size induced by the six isolates on cvs. Paraguayo and Dormilón showed statistically significant differences among treatments (*p* ≤ 0.05). However, the largest lesion size was observed in cv. Dormilón; these results are consistent with field observations, as the most severe symptoms were recorded in this variety during plant collection. Furthermore, isolate CCLF674 induced the largest lesion size in both cultivars, whereas isolate CCLF675 produced the smallest lesions in both cultivars (Table 2).

### 3.5. Identification of Antagonistic Fungi

**Figure 5:**
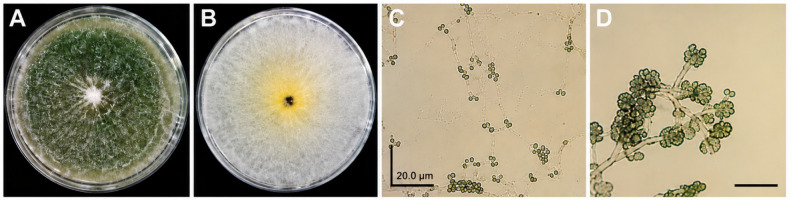
Morphological and cultural characteristics on PDA medium of *Trichoderma* isolate FAVF675. (**A**) obverse. (**B**) reverse of colony. (**C**) conidia. (**D**) phialides with conidia.

Both colonies exhibited morphology consistent with the genus *Trichoderma*; isolates FAVF675 (Figure 5) and FAVF676 showed abundant sporulation on the obverse and white coloration on the reverse of the plate. Isolate FAVF675 had a mycelial growth rate of 23.42 mm día-1, globose conidia (3.95 × 3.33 µm), short phialides (3.044 µm), and angles of 43.5°. Strain FAVF676 had a growth rate of 22.91 mm día-1, globose conidia (3.61 × 3.18 µm), and phialides 2.95 µm in length with apertures of 42.3°. Sequences PZ457791 and PZ457792 showed 100% similarity to strain MH911417 of *T. asperellum*. However, further analyses are required to confirm the phylogenetic species. Furthermore, the three isolates H7, H21, and H27 exhibited morphologies consistent with the genus *Trichoderma*, and the sequences PZ457793-PZ457795 identified the isolates as members of the genus *Trichoderma.* However, further analyses are also required to confirm the phylogenetic species.

### 3.6. Antagonism Assays

**Figure 6:**
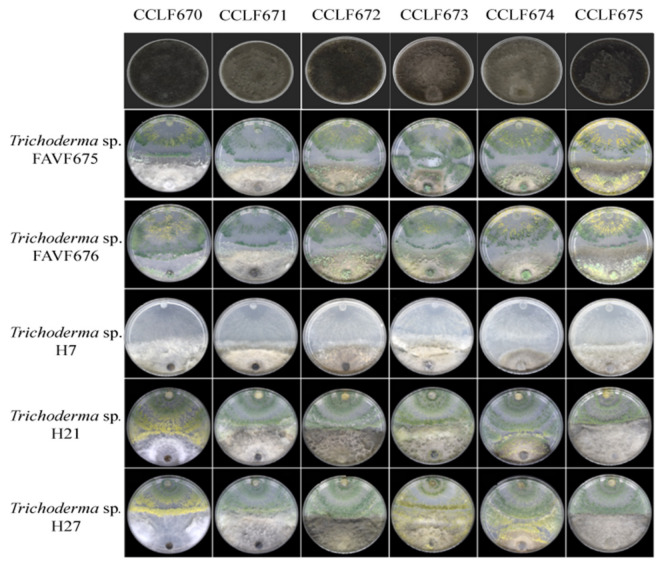
Dual cultures of *Trichoderma* spp. isolates against *Macrophomina phaseolina* isolates (CCLF670-CCLF675). In each confrontation, *Trichoderma* isolates are positioned at the top of each Petri plate, while *M. phaseolina* isolates are placed at the bottom.

Dual culture assays between *Trichoderma* spp. and the six *M. phaseolina* isolates (Figure 6) showed antagonistic activity with significant differences (*p* ≤ 0.05) in percent mycelial growth inhibition (PMGI) (Table 3). The isolate FAVF676 induced the highest inhibition against *M. phaseolina* isolate CCLF674 (Figure 5). PMGI values for FAVF675 against the six *M. phaseolina* isolates differed significantly (*p* ≤ 0.05), with the highest inhibition (60.9%) observed against CCLF674. Meanwhile for H21, the highest PMGI was also observed against isolate CCLF674, showing significant differences (*p* ≤ 0.05) with CCLF671 but not with the other isolates. Likewise, H27 showed the highest inhibition against CCLF674.

### 3.7. Fungicide Sensitivity Results

**Figure 7:**
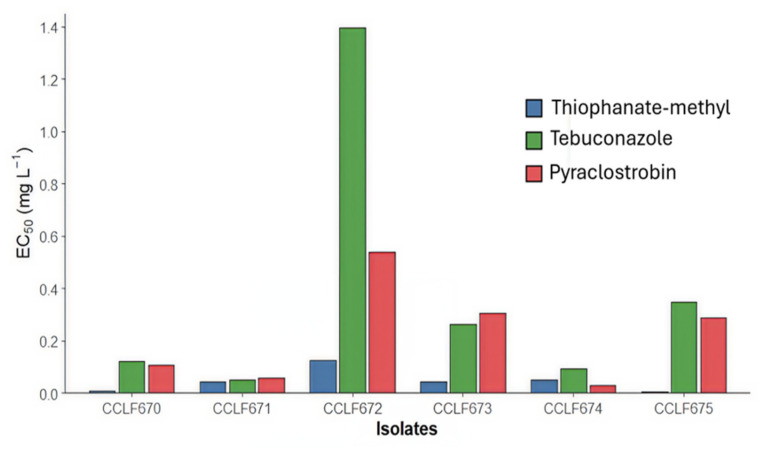
Effective concentration at which thiophanate-methyl, tebuconazole, and pyraclostrobin inhibit 50% of the mycelial growth of six *Macrophomina phaseolina* isolates infecting sesame plants.

**Figure 8:**
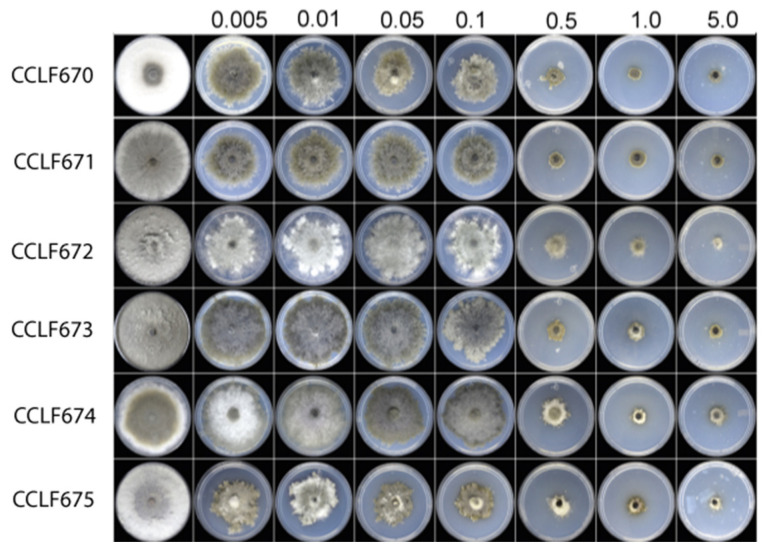
Effect of the fungicide tiophanate-methyl on the mycelial growth of *Macrophomina phaseolina* isolates.

**Figure 9:**
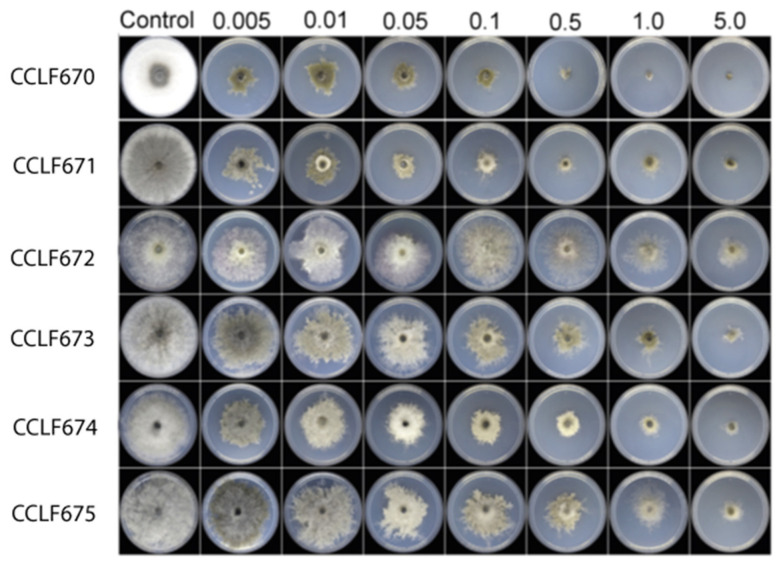
Effect of the fungicide tebuconazole on the mycelial growth of *Macrophomina phaseolina* isolates.

**Figure 10:**
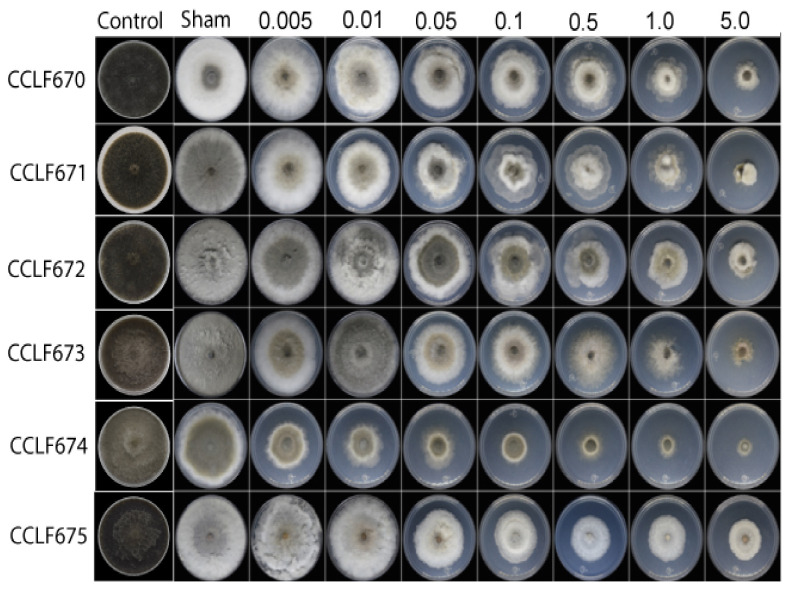
Effect of the fungicide pyraclostrobin on the mycelial growth of *Macrophomina phaseolina* isolates.

The EC_50_ values for thiophanate-methyl, tebuconazole, and pyraclostrobin ranged from 0.002–0.123, 0.049–1.397, and 0.029–0.539 mg L^−1^, respectively (Figure 7). In general, the isolates were sensitive to the evaluated molecules (Figure 8, Figure 9 and Figure 10); however, the CCLF672 isolate was the least sensitive among the three fungicides, being particularly resistant to tebuconazole.

## 4. Discussion

In this study, the causal agent of root and stem rot in sesame crops from Sinaloa, Mexico, was identified as *M. phaseolina* based on pathogenicity tests, morphological, and phylogenetic analysis. The MpTefF/MpTefR primers used to amplify the partial sequences of tef1-α are species-specific, providing taxonomic accuracy for the identification of *Macrophomina* species [20]. While multilocus analyses involving additional markers (such as act, β-tub, and cal) provide comprehensive genetic profiles, previous studies have demonstrated that the combination of ITS and tef1-α sequences yields sufficient resolution for the accurate discrimination of *Macrophomina* species [15,19,20,42].

Wilting, root, and stem rot symptoms are typically associated with a complex of soilborne fungi in northern Sinaloa production areas, where recurrent cropping of chickpea, bean, maize, oilseeds, and vegetables promotes pathogen persistence [15,42,43,44,45]. In chickpea, soilborne fungal complexes include *Fusarium* spp., *Neocosmospora falciformis*, *M. phaseolina*, *Rhizoctonia solani*, *Agroathelia rolfsii, Sclerotinia sclerotiorum*, and *Clonostachys chloroleuca* [15,44,45]. In common bean, *M. phaseolina* was associated with charcoal rot in Sinaloa [42,46], while Moreno-Gallegos et al. (2017) [47] reported that charcoal rot in sorghum may cause 30–100% yield losses. Similarly, according to reports from various authors [43,44,45,46,47,48] *F. oxysporum, F. nygamai*, *M. phaseolina*, *R. solani*, and *N. falciformis* are the causal agents of tomatillo wilt.

Morphological and molecular characterization in this study confirmed that all six isolates belonged to *M. phaseolina* and were pathogenic when inoculated into sesame stems, inducing root and stem rot, chlorosis, and wilting; variations in virulence were also detected. According to Mayek-Pérez et al. (2001) [49], *M. phaseolina* exhibits considerable morphological and virulence variability, suggesting high genetic diversity even among isolates from a single host, which may be linked to pathogen evolution. Martinez and Laurentin (2012) [12] emphasized the importance of understanding sesame field histories to assess whether germplasm exchange and seed transmission contribute to the spread of *M. phaseolina*.

Among the six isolates analyzed, CCLF671 showed the highest mycelial growth rate (22.5 mm day^−1^), although the largest microsclerotia (114.0 µm) were found in isolate CCLF674; these factors may influence isolate aggressiveness. Environmental and agroecological conditions in sesame-producing regions of Sinaloa may play a key role in pathogenesis, as supported by Aboshosha et al. (2007) [50] and Marquez et al. (2021) [51], though further research is needed.

For charcoal rot management, both biological and chemical controls are potential strategies, which require further research with potted plants and subsequent field trials to reduce inoculum levels and disease severity. In our study, the five *Trichoderma* isolates (FAVF675, FAVF676, H7, H21, H27) inhibited the mycelial growth of all six *M. phaseolina* isolates by 52% (H27) to 63% (FAVF676). *Trichoderma* spp. exhibited competition for space and nutrients, and mycoparasitism. As reported by Dhingra and Sinclair (1978) [52] and Marquez et al. (2021) [51], *Trichoderma* parasitism involves degradation and collapse of the pathogen’s hyphal walls and resistance structures.

Chemical control is also an essential tool for managing soilborne pathogens such as *M. phaseolina*. In vitro fungicide sensitivity tests help assess fungal response to chemical pressure [53]. Mahmoud et al. (2006) [54] evaluated the systemic fungicide flutolanil and found EC_50_ values ranging from 1 to >100 mg mL^−1^ among 20 isolates, highlighting variability in fungicide efficacy even within the same species. Similarly, Baldiga-Tonin et al. (2013) [55] reported that carbendazim (EC_50_ = 0.23 mg L^−1^) was the most effective fungicide against *M. phaseolina* from soybean in Brazil. This result was comparable to thiophanate-methyl (EC_50_ = 0.123 mg L^−1^) in our study.

In *M. pseudophaseolina*, Negreiros et al. (2020) [56] found EC_50_ ranges of 0.013–0.089 mg L^−1^ for carbendazim, similar to our findings for *M. phaseolina* isolates from sesame, which showed EC_50_ values of 0.00288–0.12331 mg L^−1^ for thiophanate-methyl. Negreiros et al. (2022) [57] also reported *M. phaseolina* isolates from melon, weeds, and watermelon with carbendazim EC_50_ = 0.012 μg mL^−1^. Both fungicides belong to the MBC class, which are systemic agents that inhibit fungal cell division, aligning with our findings on *M. phaseolina* growth inhibition.

Regarding pyraclostrobin and *Trichoderma* effects on *Macrophomina*, Ayvar-Serna et al. (2024) [58] found, under greenhouse conditions, that *Trichoderma* spp., botanical extracts, and fungicides effectively controlled *M. pseudophaseolina* in pepper plants. These findings could serve as a baseline for further research evaluating the antagonistic fungi and fungicides tested in this study under greenhouse conditions, as a fundamental component of an integrated management strategy.

## 5. Conclusions

In conclusion, morphological characteristics (colony color, mycelial type, and microsclerotia production) and molecular identification using ITS and *tef1-α* species-specific primers, along with phylogenetic analyses, confirmed that the six *Macrophomina* isolates from sesame correspond to *M. phaseolina*. All isolates evaluated were pathogenic and exhibited varying degrees of aggressiveness. The in vitro assays demonstrated the biocontrol potential of *Trichoderma* spp., while *M. phaseolina* isolates were sensitive to thiophanate-methyl, tebuconazole, and pyraclostrobin. More research is needed to develop an integrated management strategy that incorporates tolerant cultivars, biocontrol agents, and fungicide treatments.

## Figures and Tables

**Table 1:** Cultural and morphological characterization of *Macrophomina* sp.

Isolate	Colony			Microsclerotia n = 50 (µm)
	Colour ^++^	Mycelium	TC (mm día ^−1^)	Min–Max
CCLF670	Black	Dense	20.5 ± c	62.6 d (53.7–78.5)
CCLF671	Grey	Subfloccose	22.5 ± ab	110.2 ab (95.3–126.8)
CCLF672	Black	Dense	18.9 ± a	103.0 b (83.3–133.8)
CCLF673	Grey	Floccose	15.7 ± a	72.7 c (56.7–124.4)
CCLF674	Grey	Subfloccose	21.2 ± b	114.0 a (79.9–161.9)
CCLF675	Black	Dense	19.4 ± bc	71.1 cd (54.3–92.7)

Note: Means that shares the same letter in the same column do not show significant differences. (Tukey; *p* ≤ 0.05). ++ = 7 days of incubation at 25 ± 2 °C. Color in culture medium PDA, Microsclerotia obtained in PNA medium.

**Table 2:** Pathogenicity and aggressiveness *of Macrophomina phaseolina* isolates on the Paraguayo and Dormilón sesame varieties.

Isolate	Var. Paraguayo Lesion Length (mm)	Var. Dormilón Lesion Length (mm)
CCLF670	16.8 ± 0.6 b	21.8 ± 0.7 c
CCLF671	16.7± 0.6 b	23.6 ± 0.6 b
CCLF672	11.2± 0.6 c	14.6 ± 1.1 e
CCLF673	11.4 ± 0.6 c	16.8 ± 1.1 d
CCLF674	23.2 ± 0.6 a	26.9 ± 0.7 a
CCLF675	7.9 ± 0.6 d	12.7 ± 0.2 f
Control	0.00 e	0.00 g

Means sharing the same letter within the same column do not present statistically significant differences Tukey’s HSD; (*p* ≤ 0.05).

**Table 3:** Percentage of mycelial growth inhibition of *Trichoderma* spp. isolates against six *Macrophomina phaseolina* isolates obtained from diseased sesame plants in Sinaloa, Mexico.

*M. phaseolina* Isolates	Mycelial Growth Inhibition (%)				
	*Tichoderma* sp.				
	FAVF675	FAVF676	H7	H21	H27
CCLF670	56.2 ± 0.79 c *	58.6 ± 0.45 ab	58.5 ± 0.97 a	59.9 ± 0.03 ab	52.4 ± 0.7 d
CCLF671	57.1 ± 1.55 bc	55.7 ± 0.64 b	61.1 ± 1.27 a	55.2 ± 1.29 b	52.9 ± 0.78 cd
CCLF672	59.6 ± 0.91 abc	57.3 ± 1.02 ab	62.0 ± 0.63 a	59.5 ± 1.14 ab	59.5 ± 0.64 ab
CCLF673	58.3 ± 0.33 abc	59.7 ± 1.36 ab	60.1 ± 1.93 a	57.2 ± 0.62 ab	52.9 ± 0.82 cd
CCLF674	60.9 ± 0.5 a	63.0 ± 0.72 a	62.0 ± 0.3 a	61.1 ± 1.53 a	62.8 ± 1.14 a
CCLF675	60.0 ± 0.34 ab	57.9 ± 0.57 ab	62.7 ± 1.28 a	56.3 ± 0.71 ab	56.6 ± 1.11 bc

* Means followed by the same letter within a column are not significantly different according to Tukey’s test (*p* ≤ 0.05).

## Data Availability

The original contributions presented in this study are included in the article. The nucleotide sequences generated in this study have been deposited in GenBank under accession numbers PV106184–PV106189 for ITS, PV113476–PV113481 for tef1-α, and PZ457791-PZ457795 for ITS of *Macrophomina phaseolina* and *Trichoderma* spp. isolates, respectively. Further inquiries can be directed to the corresponding author(s).
